# Supplementary material for: The Activation of JAK/STAT3 Signaling and the Complement System Modulate Inflammation in the Primary Human Dermal Fibroblasts of PXE Patients
Source: Biomedicines. 2023 Sep 29;11(10):2673. doi: 10.3390/biomedicines11102673 (PMC10603841; doi:10.3390/biomedicines11102673)
Supplement: Supplementary file 1 [file biomedicines-11-02673-s001.zip › biomedicines-2524874-supplementary.pdf]

Supplementary file

Table S1: Primer sequences used for quantitative real-time PCR

Gene	Protein	5´-3´ Sequence	Reference	Annealing Temperature (°C)	Efficiency
ACTB *Beta-actin*	ß-Actin	CGCGAGAAGATGACCC ATTGCCAATGGTGATGAC	NM_001101	59	2.0
GAPDH *Glycerinaldehyd-3-phosphate-dehydrogenase*	GAPDH	AGGTCGGAGTCAACGGAT TCCTGGAAGATGGTGATG	NM_002046	59	1.8
ß2M Beta-2-mikroglobulin	ß2M	TGTGCTCGCGCTACTCTCTCTT CGGATGGATGAAACCCAGACA	NM_004048	59	2.0
STAT3 *Signal transducer and activator of transcription 3*	STAT3	ATGGAGATTGCCCGGATTGT AGCTCACTCACGATGCTTCTC	NM_001384993.1	66	1.8
SOCS3 *Suppressor of cytokine signaling 3*	SOCS3	GGAGACTTCGATTCGGGACC GAAACTTGCTGTGGGTGACC	NM_003955.5	68	2.0
IL-6 *Interleukin-6*	IL-6	ACAGCCACTCACCTCTTCAG GTGCCTCTTTGCTGCTTTCAC	NM_000600.4	63	1.9
MCP-1 *Monocyte chemoattractant protein-1*	MCP-1	CTTCTGTGCCTGCTGCTCATAG CTTCTGTGCCTGCTGCTCATAG	NM_002982.3	63	2.0
CXCL1 *Chemokine (C-X-C motif) ligand 1*	CXCL1	TCCTGCATCCCCCATAGTTA CTTCAGGAACAGCCACCAGT	NM_001511.4	63	1.8
IGFBP3 Insulin-like-growth-factor-binding-protein-3	IGFBP3	GCGCCAGGAAATGCTAGTGA GGGAATGTGTACACCCCTGG	NM_001013398.1	63	1.8
C3 *Complement C3*	C3	GAGAAGACTGTGCTGACCCC GATGCCTTCCGGGTTCTCAA	NM_000064.4	68	1.9
C1r *Complement C1r*	C1r	GAGGAGAATGCCCAGTGGTGG GCTTCACCCTGTATCCCGTG	NM_001354346.2	68	1.9
C1s *Complement C1s*	C1s	ACCAAAGAAGGTGCTTGTGTTT AGTGTCTCCTGAGATTATCACATC	NM_001346850.2	63	2.0
C2 *Complement C2*	C2	CCTGCCCTCAGAACGTGAAT CTGGACAGCGCACAGGTTT	NM_000063.6	66	1.9
C4A *Complement C4A*	C4A	CCAAGCTGACCTCCCTCTC TAACCTGGAAGCCGTACTCC	NM_007293.3	63	1.9

CFB<br>*Complement factor B*	CFB	CCCCGGTCTCCCTACTACAA<br>AGGTGACGCTGTCTTCAAGG	NM_001710.6	66	1.8
CFH<br>*Complement factor H*	CFH	GGAAGAGGAGAACTGGACGTTG<br>ATGTCCACAGGGCCTTTTCTG	NM_000186.4	66	1.9
SERPING1<br>*Serpin family G member 1*	SERPING1	CAGAAGTTTGGAGTCCGCTG<br>TGTTGTTGCGACCTTCCCTT	NM_000062.3	68	2.0

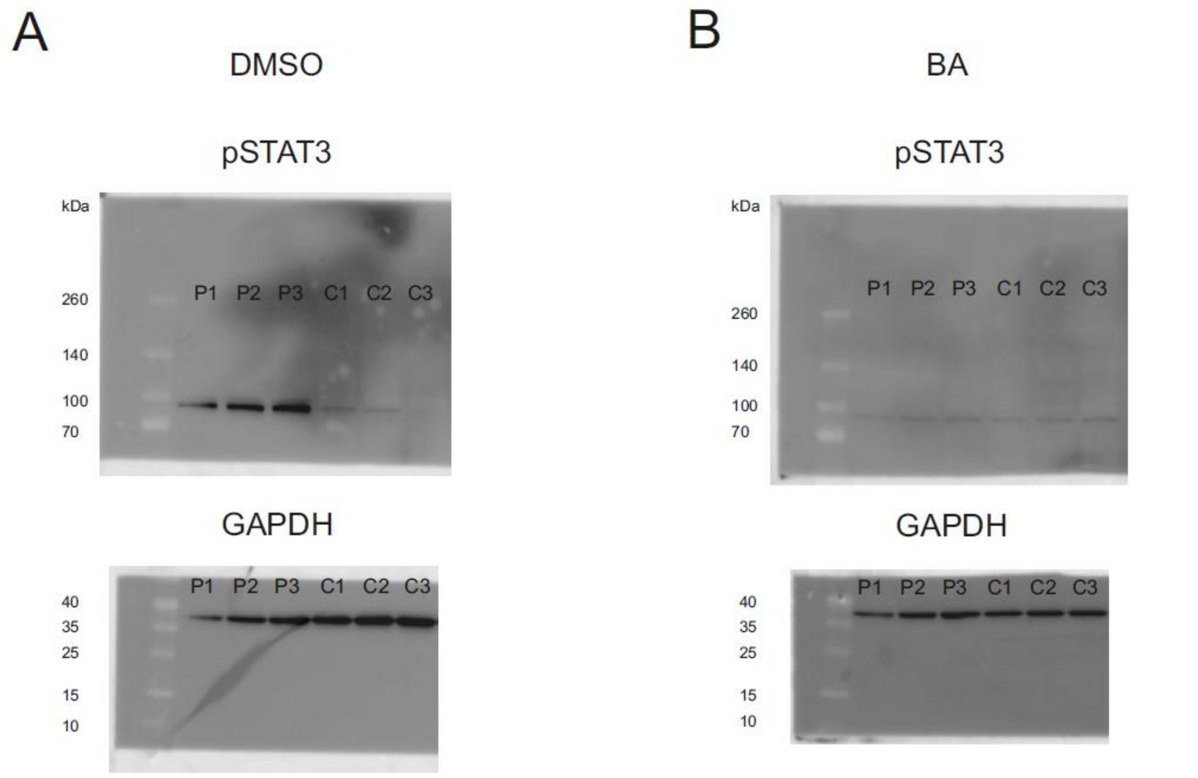

Figure S1: Western blot analysis of PXE- and control fibroblasts of pSTAT3 and GAPDH. Dermal fibroblasts from patients with pseudoxanthoma elasticum (P1-3) (n = 4) and normal human dermal fibroblasts (C1-3) (n = 4) were cultivated for 72 h in medium with 10 % lipoprotein deficient fetal calf serum (LDPS. Fibroblasts were treated with (A) DMSO or (B) 1 µM baricitinib (BA). Active STAT3 phosphorylated at tyrosine 705 (pSTAT3) and GAPDH were analyzed, 20 µg protein were used for analysis.
